# Carbon monoxide exposure in pregnant women in the UK

**DOI:** 10.1186/s12884-025-08126-6

**Published:** 2025-10-10

**Authors:** Elsie Place, Hilary Wareing, Mari Herigstad

**Affiliations:** 1 Biomolecular Sciences Research Centre, Sheffield Hallam University, City Campus (Owen Building), Sheffield, UK; 2 Bateson Centre, University of Sheffield, Sheffield, UK; 3 Improving Performance in Practice, Warwick, UK

**Keywords:** Carbon monoxide, Pregnancy, Air pollution

## Abstract

**Background:**

Carbon monoxide (CO) is a colourless, odourless gas that poses a threat to life at concentrations of just a few hundred ppm. The developing foetus is particularly vulnerable to CO exposure, and maternal exposure to much lower levels of the gas is associated with adverse outcomes such as low birth weight. This study aimed to quantify CO exposure in pregnant women’s homes and assess whether breath CO levels could be linked to home-based CO exposure and sociodemographic factors.

**Methods:**

CO levels were monitored continuously over two weeks in 161 households selected for indicators of lower socio-economic status and proximity to gas appliances, a risk factor for environmental CO exposure. Exhaled breath CO measurements were taken before and after the monitoring period.

**Results:**

Of the households monitored, positive CO readings were detected in 57.8%, with 31.7% recording levels above 4ppm and 14.3% above 10ppm. CO exposure varied significantly across households, with both intermittent and prolonged exposures observed. Six households included in the study exceeded current World Health Organisation recommended limits of 3.5ppm for ≥ 24 h, and three exceeded the limit of 9ppm for ≥ 8 h. Higher CO levels in the household were associated with the use of gas for cooking. Higher exhaled CO levels were associated with number of smokers in the household and eligibility for the UK government NHS Healthy Start scheme. Following the monitoring period, exhaled CO levels were only associated with number of smokers in the household, suggesting an intervention effect.

**Conclusions:**

This study indicates that exposure of pregnant women to CO within the home occurs predominantly within current recommended safe limits, and that exposure is linked to the use of gas appliances, socio-economic factors and smoking. This study highlights the need for improved CO monitoring and mitigation strategies, particularly in vulnerable populations, to protect maternal and foetal health.

**Supplementary Information:**

The online version contains supplementary material available at 10.1186/s12884-025-08126-6.

## Background

Carbon monoxide (CO) is a colourless, odourless, and tasteless gas that presents a health risk to exposed individuals. CO binds readily to haemoglobin in the blood, forming carboxyhaemoglobin (COHb) at the expense of oxyhaemoglobin (OHb) [1], and also shifting the OHb dissociation curve, leading to potentially lowered oxygen release to tissues and therefore hypoxia [2–4]. Toxic effects beyond hypoxia may also contribute to ongoing morbidity, as symptoms may persist or emerge even after COHb levels normalize [5]. Acute CO poisoning in pregnant women is linked to complications such as preterm birth and miscarriage, with the outcomes of pregnancy generally being influenced by the severity of maternal poisoning and the stage of foetal development [6]. However, the developing foetus is uniquely vulnerable to disruptions, and at elevated risk of CO poisoning due to foetal Hb having a higher affinity for CO than adult Hb, a risk that continues into the neonatal period as foetal Hb persists for approximately 6 months after birth. Case reports of maternal CO poisoning are fortunately rare, but highlight a range of serious adverse foetal outcomes, including cerebral palsy [7], hypoxic ischemic encephalopathy [8, 9] and cardiomegaly [10] as well as death [8].

Chronic exposure to subacute levels of CO – particularly in the context of maternal smoking – is also associated with a range of adverse foetal outcomes [11–17] including foetal growth restriction and low birth weight. Animal studies have shed further light on CO effects, showing that exposures leading to maternal COHb levels associated with smoking (75-150ppm) lead to behavioural and brain histochemical abnormalities in offspring [18–22], and recently linking low (≤ 18ppm) CO levels to changes in the developing heart [23]. Even maternal exposure to second-hand smoke has been associated with impaired motor ability [24] and neurodevelopmental delay [16]. Lee et al. furthermore observed a decrease in mental developmental index score (which incorporates attentiveness and response to stimulation) in children (6 months) whose mothers had been exposed to second-hand smoke during pregnancy, even after adjusting for covariates such as residential area, maternal age and education, income and birth weight [16]. Epidemiological associations between low birth weight and environmental CO increases of 1.4ppm [25], and levels of 5.5ppm and above [26], have been reported. Maternal chronic exposure to CO through woodsmoke at levels of 12.5ppm and below has also been linked to lower neuropsychological test scores, visuo-spatial integration and motor performance in children aged 6–7 years [13]. Therefore, there is some uncertainty regarding safe CO levels for the unborn child. Reflecting the potential for harm at even single digit concentrations, current safety guidelines state that individuals should not be exposed to an excess of 9 ppm for 8 h (or more), or 3.5 ppm for 24 h (or more, WHO, 2020 [27]), substantially lower than the levels generally associated with toxic symptoms in adults.

In 2010, the National Institute for Health and Care Excellence (NICE) issued guidelines recommending the use of exhaled CO as an indicator of smoking in pregnant women [28]. Consequently, midwives became involved in monitoring CO levels in pregnant women, leading to an observation that elevated breath CO levels are present in a subset of non-smoking pregnant women. This suggested that these women were exposed to alternate sources of CO, potentially including the home and/or urban environment, and that there remains uncertainty regarding the scale of CO exposure among pregnant women and therefore its potential impacts. To address these issues, this study sought to quantify CO levels in the homes of pregnant women through a two-week monitoring period and to assess whether breath testing at booking could serve as an effective indicator of home-based CO exposure.

## Methods

Participants were recruited from maternity services at the University Hospital Coventry & Warwickshire, Royal United Hospital Bath, Queen Elizabeth Hospital Gateshead, Mid Yorkshire NHS Trust (Pinderfields and Pontefract Hospitals) and Northumbria Hospital. Study sites were chosen to include those serving deprived populations, as women from such areas might have less access to CO monitoring equipment and may experience higher levels of exposure due to suboptimal housing conditions and appliance malfunctions. Pregnant women aged 18 years or older, presenting for care at a booking visit or in the early stages of their pregnancy, were eligible for the study. The inclusion criteria were: pregnant; currently living in private rented accommodation or social housing or eligible for healthy start vouchers; living in a property with gas heating/cooker and/or solid fuel or oil and/or living above a takeaway/café/restaurant; able to provide informed consent for the study. Healthy Start vouchers is a scheme to support low-income families in the UK, with eligibility based upon receipt of additional support due to low household take-home pay, and it can thus be used as a marker for low income. Women from all ethnic backgrounds were considered and interpreter services were available for the consent and data collection process to aid inclusivity. Household data was collected to identify CO sources other than smoking. Ethical approval was obtained under Health Research Authority/Health and Care Research Wales (IRAS ID 301261). Informed consent was obtained from all participants in the study.

### Protocol

The study included two experimental home visits (outlined below) conducted by the participant’s local Fire and Rescue Service (FRS) and a two-week sampling period.

### Visit 1

A CO alarm (AICO Ei208, AICO, Shropshire) was fitted in the property (if one was not already present) as a safety measure. An expired breath CO test was obtained from the participant using Bedfont Toxco Breathalysers (Bedfont Scientific Ltd, Kent, England) according to the manufacturer’s instructions. CO data loggers (EL-USB-CO, Lascar Electronics, Wiltshire, UK), which measure and store over 32k CO readings over a 3 to 1000 ppm range, were placed in each property at the area of the highest perceived risk. Risk was subjectively assessed by trained FRS personnel and took into account factors such as ventilation, presence of gas appliances, and height in room.

### Sampling period

The CO data logger remained in the property for a minimum of two weeks, recording ambient CO (one reading every 5 min).

### Visit 2

Following the two-week sampling period, the dataloggers were collected by the FRS and the anonymised data downloaded. During this visit, the maximum CO level measured by the CO alarm was collected to determine if it had activated, and a further breath test was taken. A questionnaire (collected manually or using a laptop) was completed by the FRS using information provided by the pregnant women and observations made whilst in the home. The questionnaire covered household details including form of housing and residents’ status, as well as the presence of any appliance(s) and fuel type(s) [29]. Women were asked about the number of smokers in the household but were not asked whether they personally were smokers. Table 1 outlines all parameters recorded in this study.

**Table 1:**
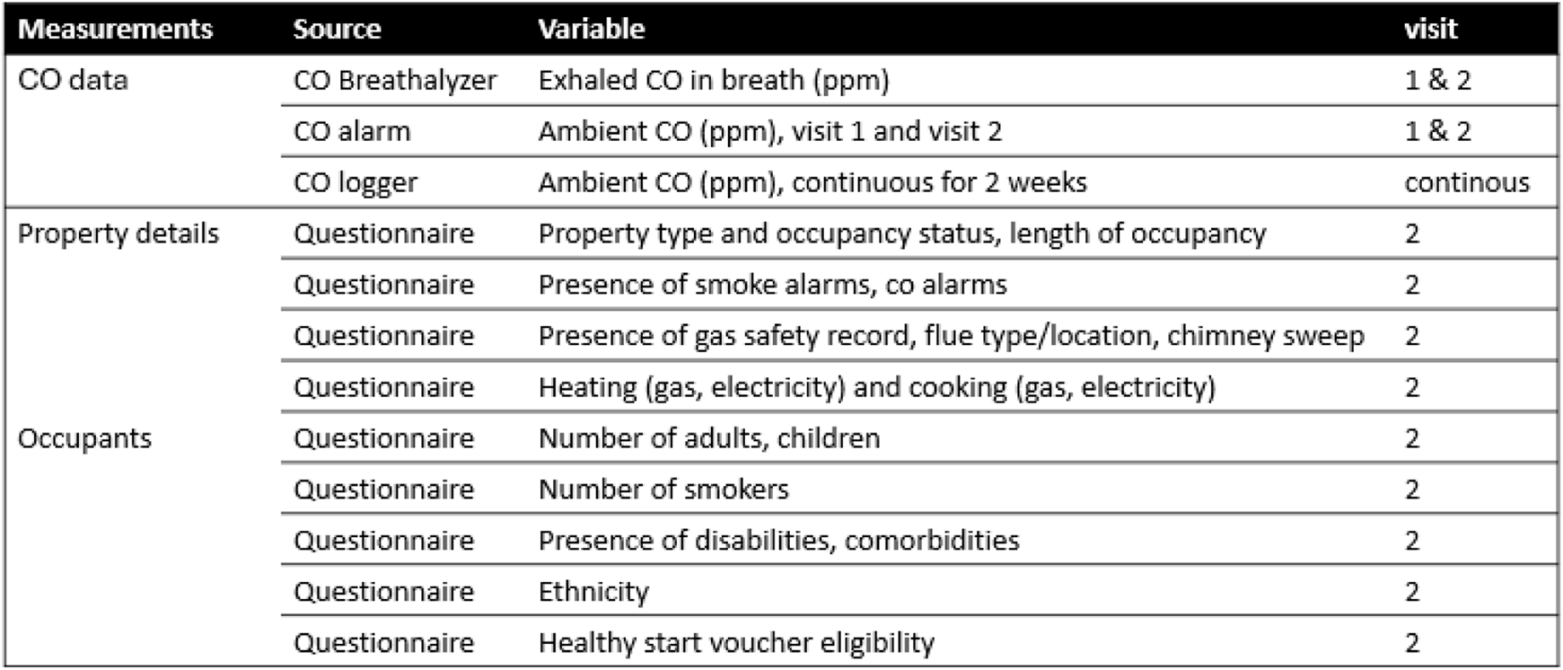
Data collected, sources and variables

### Data analysis and statistical comparisons

The readings from the data loggers (CO timeseries data) were downloaded using EasyLogUSB, Lascar’s own software (Lascar Electronics, 2021), and converted to.txt files with a unique participant identification number. All CO logger files were then truncated at two weeks, to ensure uniform sampling time. No file was shorter than two weeks. All other data was collated on a spreadsheet (Microsoft Ltd, Redmond, US) using the same identification code.

Timeseries data was analysed using custom-written software (MATLAB, Mathworks Inc, Natick, US) to quantify overall maximum (max) CO exposure levels, overall average (mean) CO exposure levels, and characteristics of any prolonged CO exposures (long exposure, LE). LEs were defined as CO above zero at each sampling point for 10 min or more (i.e. two consecutive readings above zero or more). Characteristics of LEs included total duration of LEs (in minutes), max LE duration (in minutes), mean LE duration (in minutes), and max and mean CO levels during LEs.

Timeseries data was also analysed for mean and max CO exposure levels across time of day, to generate a profile of CO exposure fluctuations on an hour-by-hour basis. This was done by averaging the CO levels across each hour (e.g. 01:00–01:55) for each individual household and assigning them to the hour for all days, then averaging across the two-week logging period. This method is a conservative assessment of fluctuations, as it did not take into account any behavioural factors, such as different routines during the week versus weekend. Outputs were visually inspected for any trends and a curve estimation was conducted (IBM SPSS Statistics, Version 27).

A Shapiro-Wilk test of normality was done on all variables, showing that none of the variables were normally distributed. CO breath measurements were compared before and after the sampling period (at first and second visit) using a Wilcoxon Rank test. An explanatory correlation analysis (IBM SPSS) was conducted to compare CO exposure levels (derived from CO timeseries data), other CO measurements obtained (breath measurements), and all demographic data (bivariate correlation). The purpose of this analysis was to identify associations between CO measurements and predictor variables, but not between individual predictor variables. A subsequent stepwise linear regression analysis was conducted to identify the most significant predictors of the CO level at visit 1 (exhaled breath) from the variables (independent variables). The criteria for entry and removal of variables were based on a significance level of 0.05. Variance Inflation Factors for all predictors were accepted if below 2. This was conducted to ascertain whether exhaled CO in the sample could be predicted by any risk factors for exposure. A stepwise regression was used as this analysis was exploratory. However, as it is prone to Type 1 error, a standard regression was also conducted. Variables included in both regression analyses were type of occupancy, ethnicity, disability status, comorbidity status, number of smokers in the household, type of cooking, eligibility for healthy start vouchers, gas safety record and season (spring, summer, autumn and winter), as well as CO measurements (overall maximum CO exposure levels, number and duration (average and median) of LEs, mean CO levels during LEs and total CO exposure).

## Results

Only complete data sets were included in the analysis, consisting of CO timeseries data, questionnaires, and breath CO and CO alarm measurements from visits 1 and 2. Complete datasets were collected from 161 participants (Table 2).

**Table 2:**
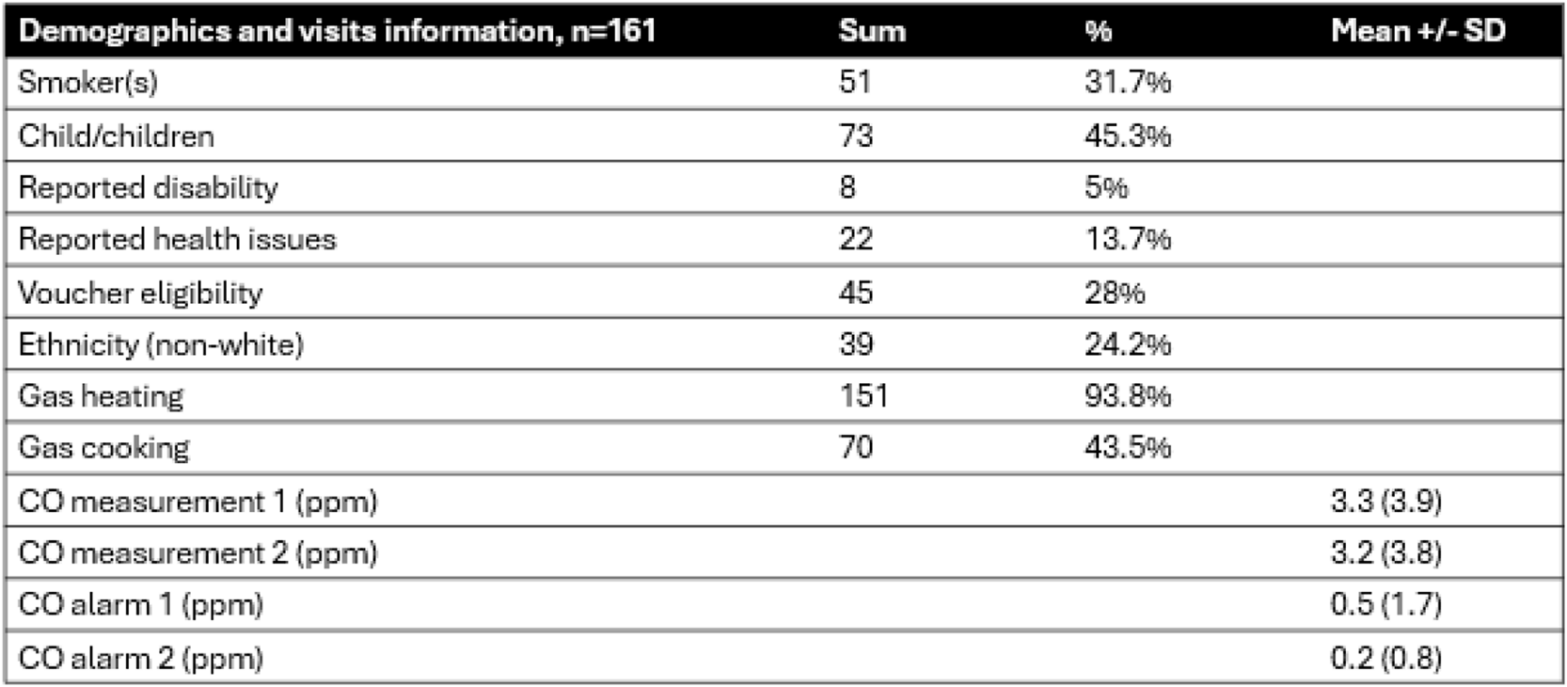
Data from visits 1 and 2 (*n* = 161)

### Demographic and household information

31.7% of households included at least one smoker, 45.3% included children, 5% included individuals with disabilities, and 13.7% included individuals with reported health issues (not specified). 28% were eligible for healthy start vouchers, and 24.2% were of non-white ethnicity. 46.6% lived in social housing. 93.8% used gas for heating as opposed to non-gas sources, and 43.5% used gas for cooking, as opposed to non-gas sources. There was no difference in CO breath measurements (Z=−0.685, *p* = 0.49) before and after the sampling period. The CO alarm data showed that none of the CO alarms had activated.

### CO timeseries analysis

**Fig. 1:**
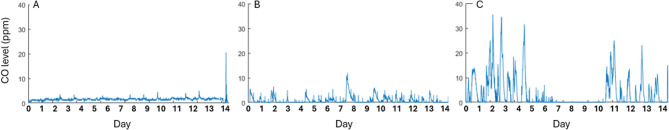
Example CO timeseries data across 14 day assessment periods, showing (**A**) chronic, low-level exposure (< 3.5ppm), (**B**), intermittent moderate exposure (< 9.0ppm) without a clear pattern and (**C**) intermittent, high exposure (> 9.0ppm) with a clear pattern

Representative CO traces highlighting different patterns of exposure are presented in Fig. 1. In some households, chronic low-level CO was measured across the monitoring period (Fig. 1A). In others, the exposure was more intermittent with either no discernible pattern (Fig. 1B) or a clear pattern of exposure (Fig. 1C). Levels varied greatly both across and within households, with some experiencing repeated or continued exposures of 10ppm and (considerably) above (Fig. 1C).

**Fig. 2:**
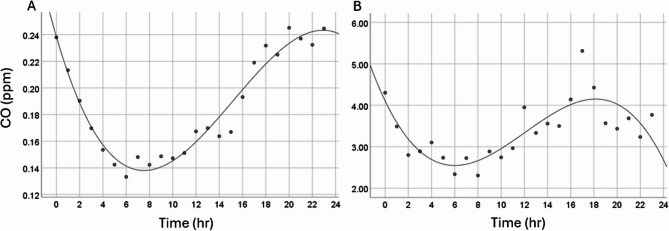
Mean (**A**) and max (**B**) CO values over the 24-hour period. Data are averages for each hour across two weeks, pooled across all samples. Data is fitted with a polynomial trendline for mean (R2 cubic = 0.95, *p* < 0.001) and max (R2 cubic = 0.81, *p* < 0.001)

Further assessment of the timeseries showed that CO levels typically were lower during the day than the night (Fig. 2A), and that there was a spike in CO levels around 17:00–18:00 (Fig. 2B). A polynomial cubic model was the best fit for the data.

### Maximum and average CO levels, and exposure duration

CO timeseries analysis showed that 57.8% of households (93 households) had at least one CO reading above zero at some point during the sampling period. 31.7% of the households experienced CO levels above 4ppm at least once (51 households), 16.7% recorded CO levels above 8ppm and 14.3% recorded CO levels above 10ppm at least once (27 households and 23 households, respectively). On average, for those with readings above zero, the time spent > 4ppm was 9 h (544.2+/−1543 min), >8ppm was 4 h (237.4+/−718.8 min) and > 10ppm was 3.3 h (198.0+/−606.3 min, not necessarily consecutive exposure) over the two-week period. 47.8% of all households had CO readings above zero that lasted 10 min or more (LEs). Three households exceeded the WHO exposure guideline of 9ppm over 8 h and six households exceeded the WHO exposure guideline of 3.5ppm over 24 h during the course of the study.

In households with CO readings above zero, the average exposure across the entire two weeks sampling period was 0.3+/−0.7ppm and the average maximum was 10.4+/−33.5ppm. Households with LEs were on average exposed 23.5 times to LEs, with a mean duration of such LEs being 533+/−2345 min and a median duration of 55 min (interquartile range (IQR) of 100 min: 33 min (Q1) −133 min (Q3)). The average CO level during LEs was 1.5+/−2.3ppm.

### CO exposure correlation analysis

**Fig. 3:**
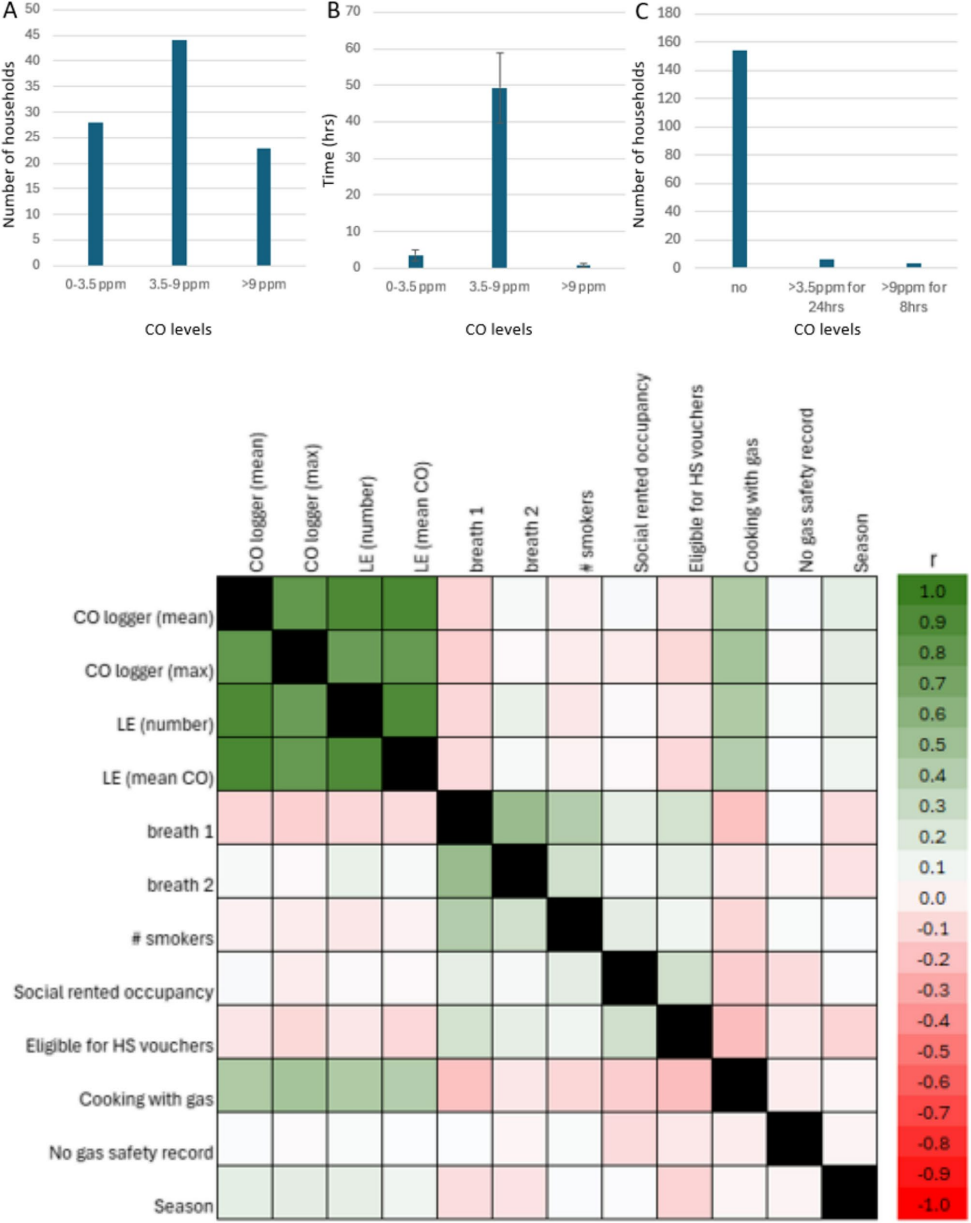
CO data. (**A**) Number of households with CO logger data showing CO exposures at 0-3.5ppm, 3-9ppm and > 9ppm at least once during the study period; (**B**) average time (not necessarily consecutive) at CO exposures of 0-3.5ppm, 3-9ppm and > 9ppm, average +/- SE; (**C**) number of households adhering to or exceeding WHO guidelines; (**D**) Exploratory analysis demonstrating correlations of changes in the measured variables across the sample (full correlation matrix). Stronger correlations are highlighted in red (negative) and green (positive correlations). r = Spearman’s rho. HS = Healthy Start

Correlations for all variables are presented in Fig. 3D, and CO-related correlations significant at the *p* < 0.01 level are described in text. This is an exploratory analysis only, designed to assess direction and strength of potential associations. Mean CO levels collected by the data loggers (CO logger (mean)) correlated with maximum CO levels (CO logger (max); *r* = 0.81, *p* < 0.001), number of LEs (LE (number); *r* = 0.91, *p* < 0.001) and mean CO during LEs (LE (mean CO); *r* = 0.92, *p* < 0.001). CO logger max correlated with LE number (*r* = 0.77, *p* < 0.001) and LE mean CO (*r* = 0.79, *p* < 0.001), and LE number correlated with LE mean CO (*r* = 0.91, *p* < 0.001). Thus, CO logger parameters correlated positively with each other. Heating method yielded no data as > 90% of households used gas for heating, meaning there was too little variation in the sample for analysis. Cooking with gas correlated with all CO log data: mean CO levels (*r* = 0.43, *p* < 0.001), max CO levels (*r* = 0.47, *p* < 0.001), number of LEs (*r* = 0.43, *p* < 0.001) and mean CO levels during LEs (*r* = 0.40, *p* < 0.001).

Finally, we observed that exhaled CO levels at visit 1 (breath1) correlated strongly with exhaled CO levels at visit 2 (breath2; *r* = 0.54, *p* < 0.001), with number of smokers in the property (# smokers; *r* = 0.41, *p* < 0.001; higher exhaled CO levels with higher number of smokers) and with voucher eligibility (*r* = 0.25, *p* = 0.002; higher exhaled CO levels with being eligible for vouchers). Exhaled CO levels (breath2) correlated only with the number of smokers (*r* = 0.27, *p* = 0.001).

### Predictors of exhaled CO

The most important predictors of exhaled CO levels at visit 1 were number of smokers in the household and voucher eligibility. As CO timeseries (logger) data correlated poorly with exhaled CO data (Fig. 3), logger data were not entered into the model. The final model explained a significant proportion of the variance in exhaled CO levels (R^2^ = 0.31, adjusted R^2^ = 0.25, F(2,89) = 19.09, *p* < 0.001). Coefficients for the predictors are presented in Table 3. The Constant predictor is the Y intercept, the height of the regression line when it crosses the Y axis. Repeating the prediction analysis for breath2 showed that only the number of smokers was a predictor for exhaled CO at visit 2 (breath2).

**Table 3:**
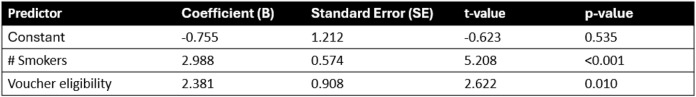
Stepwise regression analysis for breath CO measurements at visit 1

**Fig. 4:**
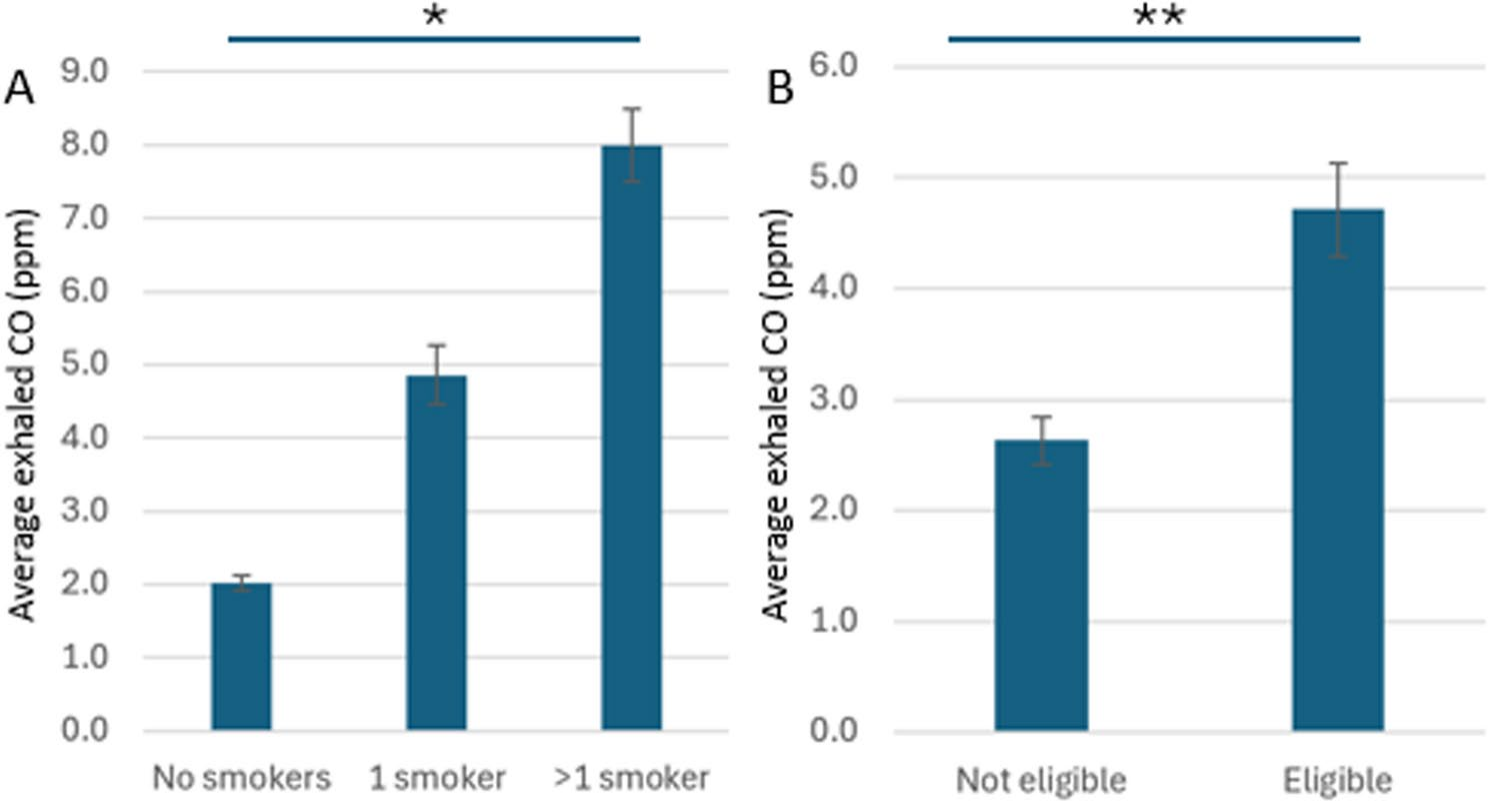
Exhaled CO (first visit) in pregnant women living in (**A**) households with no, one or more than one smoker; (**B**) households that are not eligible or eligible for healthy start vouchers. Averages +/- SE. * *p* < 0.05; ** *p* < 0.01

There was a statistically significant difference between exhaled CO levels (breath1) in households with no, one and more than one smokers (Kruskal-Wallis H test, χ2 [2] = 7.035, *p* = 0.030; Fig. 4A). There was a statistically significant difference between exhaled CO levels in households that were eligible and not eligible for Healthy Start vouchers (Mann-Whitney U test, U = 1761, *p* = 0.002; Fig. 4B).

## Discussion

This study aimed to evaluate environmental risk factors for CO exposure in pregnant women from sources other than smoking. The public health and economic burden of CO exposure remains unknown, in part because CO is not regularly assessed in the home (or, indeed, in the environment) as current safety guidelines are generally assumed to be met. Although most of the households studied were compliant with current guidelines, we detected CO in the majority of households studied, and 9 out of 161 exceeded WHO guidelines for environmental CO levels. CO levels above 4ppm and 8ppm were recorded in a third and a sixth of households, respectively, with the pattern of exposure varying greatly between households, most likely due to the underlying source of CO emission. While breath CO levels correlated with the number of smokers in the household, ambient CO levels peaked during the early evening (max) and night (mean), and correlated with the use of gas cookers, strongly implicating gas appliances as a source of exposure. For context, exhaled breath CO levels of < 5ppm, corresponding to 1.4%COHb, are typically accepted as normal.

### Exposure levels

Similar to the present findings, studies mapping CO exposure in different subsets of the UK population have indicated that CO is present in a number of households, but not necessarily at levels exceeding guidelines. In a large study of > 800 households, there were no incidences of exceeding an earlier WHO guideline of 8.6ppm, and maximum levels reported indicated peaks of 3.88ppm [30], considerably lower than the average maximum level detected here (10.4+/−33.5ppm). However, a study in East London of 270 households showed that 18% exhibited CO levels exceeding the then WHO guidelines of 8.6ppm [31]. Similarly, a study in 20 non-smoking households and 44 smoking households found that although smoking households had a higher mean (0.1-21ppm) and maximum exposure level (1.9-53.6ppm) than non-smoking households (mean: 0.1-1ppm; maximum: 4-22ppm), neither group exhibited means in excess of 10ppm over an 8-hr period [32]. In this case the sample was selected on the basis of age, which might limit potential comparisons with our study. Nevertheless, it is interesting to observe that maximum exposures were on par with those identified here.

### Source of CO

Likely sources of CO in the home are cigarette smoking and gas appliances, the presence of which might more than double the CO levels in the home [27, 33]. We found that CO levels fluctuated across the 24-hr period, both mean and max values varying with a polynomial (cubic) fit. Levels were typically higher at night, with max values also rising around 4pm-6pm. We cannot formally test the cause of these fluctuations, as participants were not asked to record their day-to-day activities, but smoking is unlikely to be the main driver for these fluctuations given their elevation at night. A more likely scenario is that CO levels are linked to gas heaters (heating turned on as the temperature drops in the evening, remaining on until the morning; heating turned off during the day as people may be at work) and gas cooking (maximum CO levels were around 17:00–18:00), both appliances known to emit CO. Importantly, we did see a significant correlation between using gas for cooking and all measured CO values in the home in the present study, which greatly supports the above interpretation and supports a similar finding from an earlier study [30]. It is also important to note that CO concentration in the immediate vicinity of the cooker will likely be higher than that detected by the loggers, and traditionally, women are more likely to be involved in food preparation, hence more at risk of exposure.

While we could not assess correlation between gas heaters and the other variables in this study, due to too few households not being heated by gas (reflecting our inclusion criteria), it is important to note that this does not rule out an impact of gas heating on our measured CO levels. Indeed, CO exposure may vary with seasonal change, being typically higher during colder months, which could in part be associated with heating. While the study was not designed to assess the impact of seasonal variation, season was included in the exploratory correlation analysis and showed significant relationships with both mean and max CO levels, with higher levels being linked to the colder seasons at the *p* < 0.05 level.

### Risk factors

 We observed correlations between exhaled CO levels at visit 1 and several factors, including maximal CO exposure (as measured by the CO data loggers), voucher eligibility and number of smokers in the property, type of occupancy and ethnicity. Exhaled CO levels remain a simple way of assessing CO exposure in pregnancy, and linking this to risk factors may thus help to identify vulnerable sections of the population. Indeed, both voucher eligibility and number of smokers were significant predictors for CO levels measured in the exhaled breath. Taken together, this supports other studies suggesting increased risk of CO exposure for deprived areas. For example, in a study on low-income households, fuel poverty was shown to be a risk factor for elevated CO levels in the home [34].

A key aim of our study was to ask whether breath CO measurements could indicate environmental exposures other than smoking. Notably, breath measurements did not emerge as a correlate of CO indices collected by the CO data loggers, nor did household CO levels predict CO breath levels. As household CO levels in the study were generally well within recommended limits, it is possible that CO breath tests may only be suitable for detecting exposures exceeding current safety guidelines. CO exposures outside the home (which we did not record) may also have introduced further variation into our measurements, confounding our analysis. Indeed, there is no straightforward translation between exposures and CO concentration in the body, although there have been several attempts at generating an algorithm for CO uptake [35]. Despite this result, it is important to note that elevated CO in a breath test cannot be presumed to be caused by exposure to tobacco smoke.

CO exposure is difficult to diagnose without a source of the gas being identified, as the gas typically causes non-specific symptoms such as dizziness, nausea and headache. Depending on the exposure level, the duration of the exposure, and the specific physical condition of the person exposed, CO could cause health implications not only in the short term, but also persisting over time [6, 36, 37]. Indeed, evidence is starting to emerge that even very low exposures may impact on health, particularly in vulnerable populations such as the developing foetus [23, 38–43]. It is thus imperative to improve our understanding of risk factors for, and prevalence of, CO exposure.

### Limitations

Recruitment was conducted from maternity services at University Hospital Coventry & Warwickshire, Royal United Hospital Bath, Queen Elizabeth Hospital Gateshead, Mid Yorkshire NHS Trust (Pinderfields and Pontefract Hospitals) and Northumbria Hospital. While no selection for socioeconomic factors was conducted during recruitment, these hospitals do serve deprived populations, which may skew thes findings towards higher CO exposures. Future studies might want to include a greater variety of study sites to assess differences in sociodemographic factors with greater accuracy. The CO loggers used in this study are designed to operate between 3-1000ppm, which is on par or better than most devices currently available for CO monitoring. While we find these CO loggers to have a good level of accuracy for low CO levels during testing under laboratory conditions, we nevertheless have refrained from reporting individual data due to potential logger measurement variation, and rather focused on averages for the analysis. No data on household ventilation or nearby traffic was included. These can impact indoor CO levels, and future studies should endeavour to incorporate such measures. Personal smoking history was not obtained as part of this study, due to the sensitive nature of this question, which constitutes a major limitation given the potential impact of this explanatory variable. While the overall number of smokers in the household was identified – a number which might capture maternal smoking as well as second-hand smoke – this is a proxy only, and does not yield information about the specific contribution of maternal smoking on exhaled CO. Indeed, both the number of smokers in the household and the eligibility for Healthy Start vouchers may be, in part, proxies for maternal smoking, highlighting the importance of this measure. Given that the number of smokers in the household was the main predictor for exhaled CO in this study, it is clear that smoking remains an important source of CO exposure in pregnancy and also that the number of smokers (i.e. potential second-hand smoke) matters. Future studies should therefore endeavour to quantify both maternal and second-hand smoke burden, ideally through a combination of self-report and physiological measures (i.e. a biomarker such as COHb or cotinine).

## Conclusions

This study shows that the CO exposure levels in a small minority of UK households is higher than current guidelines, and that this can be linked to the use of gas in cooking. The highest of the CO levels observed are on par with doses that have produced adverse foetal outcomes both in epidemiological studies in humans and in model organisms in the laboratory. This study also shows that exhaled CO levels in pregnant women can be predicted by the number of smokers in the household and eligibility for the Healthy Start scheme, but that the latter association may potentially be modified (possibly by CO awareness) as it was no longer present at the second visit. In conclusion, we argue that CO exposure remains a healthcare challenge in the pregnant population, which could have tangible public health consequences, and that further study as well as awareness campaigns are warranted, particularly in at-risk populations, to address this issue.

## Supplementary Information

Below is the link to the electronic supplementary material.

Supplementary Material 1

## Data Availability

The datasets used and/or analysed during the current study are available from the corresponding author on reasonable request.

## Abbreviations

CO: carbon monoxide
FRS: Fire and Rescue Service
LE: Long Exposures, exposures of CO above zero that lasted 10 min or more
NICE: National Institute for Health and Care Excellence
Ppm: parts per million
WHO: World Health Organization
