# Supplementary material for: Carbon monoxide exposure in pregnant women in the UK
Source: BMC Pregnancy Childbirth. 2025 Oct 10;25:1063. doi: 10.1186/s12884-025-08126-6 (PMC12512385; doi:10.1186/s12884-025-08126-6)
Supplement: Supplementary file 1 — Supplementary Material 1 [file 12884_2025_8126_MOESM1_ESM.docx]

Supplementary material. Carbon monoxide exposure in pregnant women in the UK.

Questionnaires at first and second (final) visit:


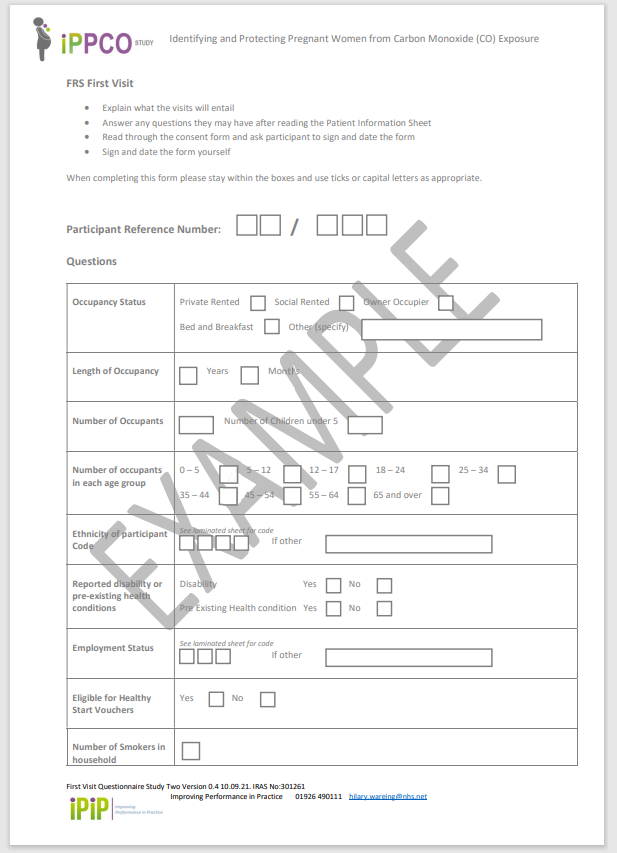


Figure S1: First visit questionnaire, page 1/4


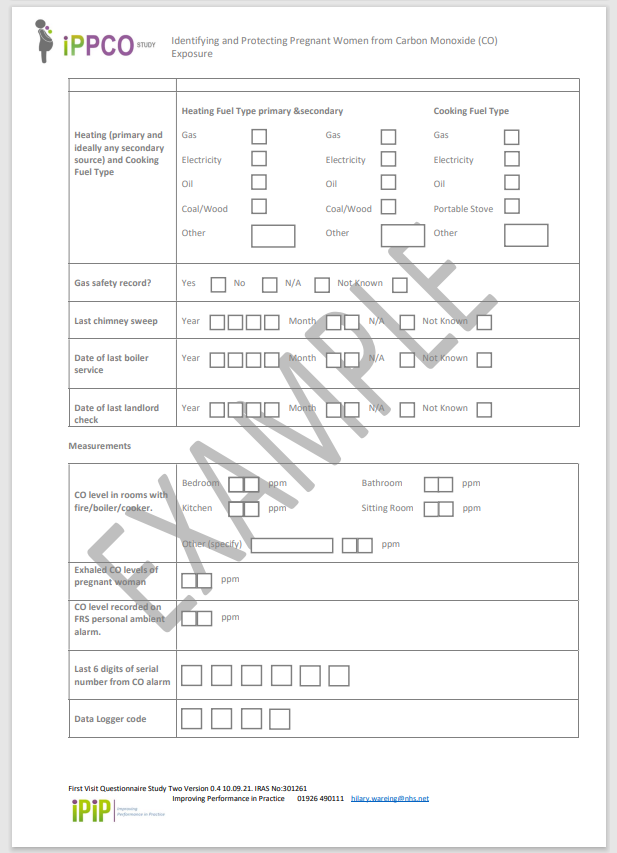


Figure S2: First visit questionnaire, page 2/4


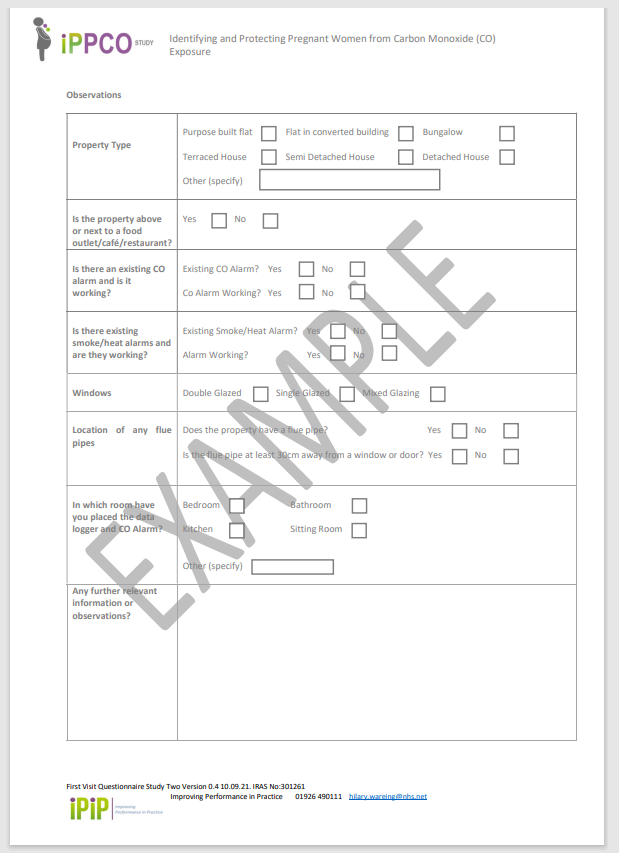


Figure S3: First visit questionnaire, page 3/4


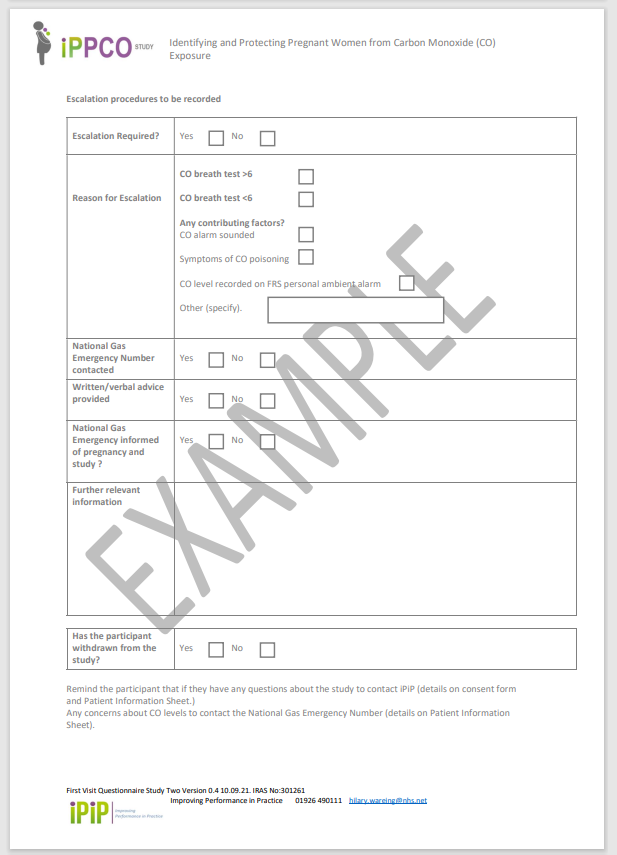


Figure S4: First visit questionnaire, page 4/4


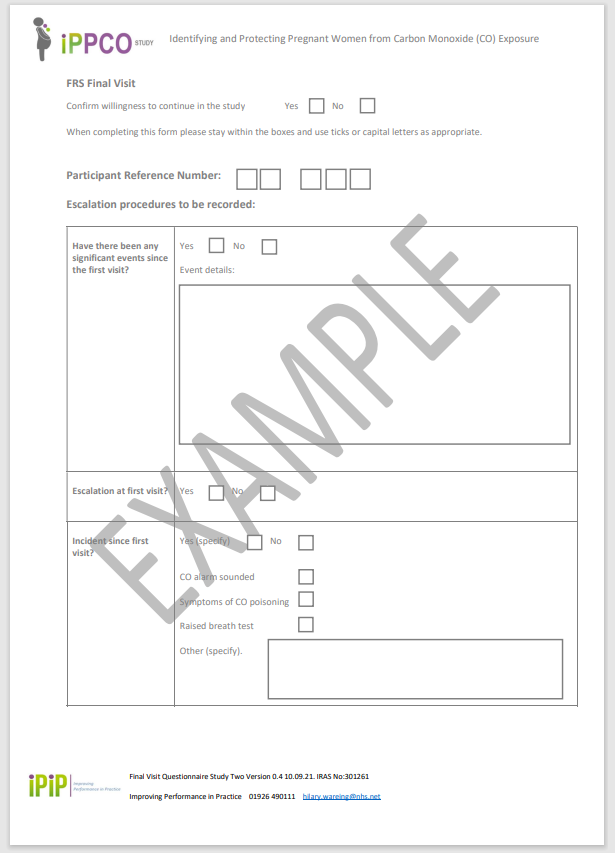


Figure S5: Second (final) visit questionnaire, page 1/3


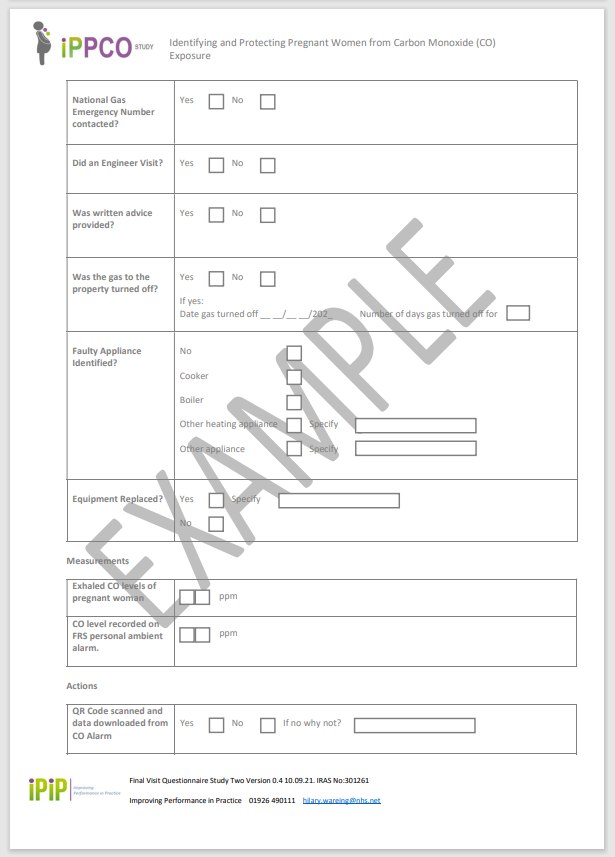


Figure S6: Second (final) visit questionnaire, page 2/3


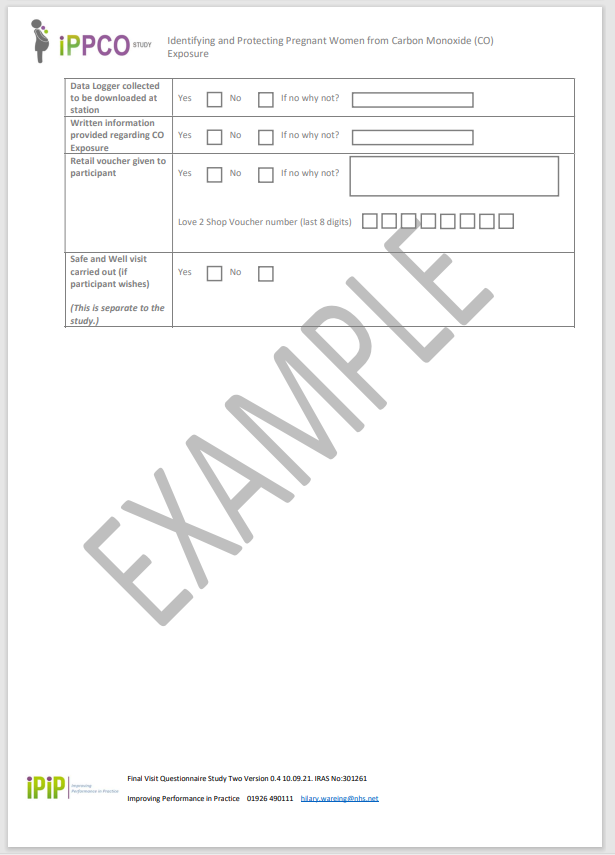


Figure S7: Second (final) visit questionnaire, page 3/3
